# Static and Dynamical Properties of heavy actinide Monopnictides of Lutetium

**DOI:** 10.1038/srep29309

**Published:** 2016-07-07

**Authors:** Showkat H. Mir, Prakash C. Jha, M. S. Islam, Amitava Banarjee, Wei Luo, Shweta D. Dabhi, Prafulla K. Jha, R. Ahuja

**Affiliations:** 1 Centre for Nano Science, Central University of Gujarat, Gandhinagar-382030, India; 2 School of Chemical Sciences, Central University of Gujarat, Gandhinagar-382030, India; 3 Condensed Matter Theory Group, Department of Physics and Astronomy, Box 516, Uppsala University, S-75120 Uppsala, Sweden; 4 Department of Physics, Maharaja Krishnakumarsinhji Bhavnagar University, Bhavnagar, 364001, India; 5 Department of Physics, Faculty of Science, The Maharaja Sayajirao University of Baroda, Vadodara, 390002, India

## Abstract

In this work, density functional theory within the framework of generalized gradient approximation has been used to investigate the structural, elastic, mechanical, and phonon properties of lutetium monopnictides in rock-salt crystal structure. The spin orbit coupling and Hubbard-U corrections are included to correctly predict the essential properties of these compounds. The elastic constants, Young’s modulus *E*, Poisson’s ratio *v*, shear modulus *G*, anisotropy factor *A* and Pugh’s ratio are computed. We found that all lutetium monopnictides are anisotropic and show brittle character. From the wave velocities along [100], [110] and [111] directions, melting temperature of lutetium monopnictides are predicted. Dynamical stability of these monopnictides has been studied by density functional perturbation theory.

In recent years, the rare earth (RE) monopnictides and chalcogenides have gained a substantial attention of solid state and material scientists owing to their diverse structural, mechanical, electronic, thermal, and magnetic properties^1^,^2^,^3^. Thin films of RE nitrides have potential application in spintronic devices due to their unique ferromagnetic behaviour and electronic properties^1^,^4^. RE monopnictides show peculiar magnetic and electronic properties correlated with partially filled *f*-shell electrons which are delocalised and strongly interact with crystal lattice^5^. This kind of behaviour have been explained in terms of mixing of *f*-orbitals with *p*-orbitals of neighbouring ion and transfer of 4*f* electrons to 5*d* conduction band of the rare earth ion^5^.

The rare earths comprise elements with atomic numbers from 57 (La) to 71 (Lu) have electronic configuration [Xe]6s^2^4f^n^, where n varies from 0 for La to 14 for Lu. Some elements (La, Gd, Dd and Lu) of RE series contain one electron in 5*d* shell also. These elements have 3+ as the most common oxidation state with 4*f* levels spanning Femi energy. They have largest orbital and spin magnetic moments due to their partially filled *f*-shell. Except promethium which has no stable isotope, they are by no means rare and are found in the earth’s crust at relatively larger concentrations than Ag, Cd, Hg, Ge, and As^6^.

The structural properties of RE monopnictides have been studied experimentally through high pressure X-ray diffraction (XRD) technique^7^,^8^,^9^. Structural phase transitions from NaCl (B1) to CsCl (B2) phase under high pressure have been extensively studied for several RE compounds^10^,^11^,^12^,^13^,^14^,^15^. In addition, the electronic band structure, lattice dynamical and elastic properties of various RE compounds have been investigated in previous studies^3^,^14^,^15^,^16^,^17^,^18^,^19^,^20^. Lattice vibrational study of RE chalcogenides SmS, Sm_x_Yb_1-x_S, TmSe and TmS_x_Se have revealed the strong electron-phonon interaction at rare earth ion site due to the hybridization of 4*f*-5*d* orbitals giving rise unusual behaviour of their phonon dispersion curves^21^,^22^,^23^. The interaction between *f* and conduction electrons varies with the interatomic distance and as a consequence the rare earth compounds have peculiar properties as a function of applied hydrostatic pressure^24^. Recently, the lutetium compounds from the group of rare earth compounds have received some attention from the researches^5^,^16^,^25^. Seddik *et al*.^25^ have performed the theoretical calculations to survey the phase transition in three lutetium compounds (LuX : X=S, Se and Te). High pressure phase transition and electronic properties of lutetium monopnictides have also been predicted by Gupta *et al*.^16^ Experimental study of the structural phase transformation of lutetium monopnictides has been reported by Shirotani *et al*.^8^

To take into account the effects of strongly correlated *f* electrons and the exchange and coulomb interactions new methods have been devised. One such method is the self-interaction correction (SIC) approach suggested by Gunnarssion and Svane^26^. But this method has certain fundamental limitations as described by Gunnarsson and Jones^27^. To correctly describe the effect of *f* electrons an alternate approach is to include Hubbard ‘U’ parameter. This, so called DFT+U^4^,^28^ approach adds a screened Hartree-Fock kind treatment for the *f*-orbitals. Therefore, in this paper, we have presented the structural, mechanical and elastic properties of some LuX (X: N, P, As, Sb and Bi) compounds using DFT+U approach with spin orbit coupling (SOC). Afterwards, employing the same scheme, phonon dispersion of these compounds is studied using finite displacement method.

## Results and Discussion

### Structural Properties

The ground state properties are obtained by optimising the lattice constant for all lutetium monopnictides using GGA+U (U = 6.0) method. Optimized lattice constants were estimated by minimizing the total energy of each structure as a function of volume. Bulk modulus is obtained from elastic constants using Eqn. (3). The optimized lattice constant (*a*_*o*_) and Bulk modulus (*B*) calculated are summarised in Table 1 along with other theoretical and experimental results for contrast and comparison. The computed lattice parameters and Bulk moduli have satisfactory agreement with experimental results and other reported theoretical data^8^,^16^,^29^,^30^. It is clear from Table 1 that the bulk modulus decreases as the radius of pnictide ion increases. Hence, the resistance to volume change of LuX decreases with increase in the size of pnictide ions.

### Elastic and Mechanical Properties

Elastic constants are related with macroscopic distortion of a crystal structure which has direct application in the evaluation of elastic energies or strains in materials under internal, external or thermal stresses^31^,^32^. The knowledge of elastic constants is important to get right view about the mechanical properties such as load deflection, internal strain, interatomic bonding, fracture, toughness, Poisson’s ratio and sound velocities of a crystal. The linear response of a crystal to external forces can be described by elastic constants which are connected with many important properties of solids like structural stability, interatomic potential, equation of state (EOS) and phonon spectra^33^. Besides, elastic constants are also related to thermodynamic properties such as specific heat, thermal expansion, Debye temperature, Gruneisen parameter and melting temperature. Therefore, determination of elastic constants is essential to characterise a solid material. In the present study, we have calculated the elastic constants of LuX using GGA+U scheme with and without SOC interactions and listed them in Table 2. Three independent elastic constants *viz*. *C*_*11*_, *C*_*12*_, and *C*_*44*_ are required to analyse the elastic properties of a cubic crystal. The elastic constant *C*_*11*_ measure the compression along the principle crystallographic axis whereas *C*_*44*_ measure the resistance to shear deformation across (100) plane in the [010] direction. We found that the elastic constants computed without SOC does not show noticeable variation when SOC was included. Therefore, elastic constants for LuN were calculated without SOC interaction. Thus, now onwards we will discuss the elastic and mechanical properties of LuX obtained using GGA+U+SOC except for LuN for which only GGA+U calculations have been done. It can be noticed from Table 2 that *C*_*11*_ is larger than *C*_*44*_ for all lutetium monopnictides which infers that these materials show weaker resistance to pure shear deformation than the unidirectional compression resistance. Furthermore, the criterion for mechanical stability of a cubic crystal is that the stress energy must be positive. This means that the elastic constants must satisfy the following conditions for mechanical stability of a crystal.


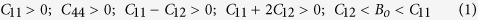


From Table 2, it is clear that the elastic constants of LuX compounds satisfy all these conditions demonstrating the mechanical stability of these compounds. The Zener anisotropy factor *A* defined by Eqn. (2) measures the degree of anisotropy in a solid^34^. For an isotropic material, *A* = 1 whereas any value other than one indicates to anisotropy of a material. Thus, anisotropy of a material is measured by simply subtracting the value of *A* from unity. As shown in Table 2, the calculated anisotropy factor (*A*) differs from unity which indicates the elastically anisotropic behaviour of LuX.


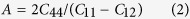


The mechanical properties such as Young’s modulus (*E*), shear modulus (*G*) and Poisson’s ratio (*v*) of LuX compounds are obtained from elastic constants employing Voigt-Reuss-Hill approximation^32^ given in Eqns (3, 4, 5, 6, 7, 8).


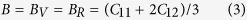



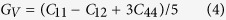



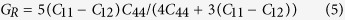



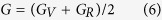



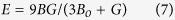



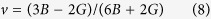


The subscript v and *R* stands for Voigt and Reuss notation respectively. Results obtained for *E*, *G* and *v* have been assembled in Table 2 together with the anisotropy factor. The constants *G* and *E* are essential to describe the stiffness of an isotropic material. It is clear from the calculated values of *G* and *E* that these materials are stiff and their stiffness decreases as the radius of pnictide ion increases. The high stiffness of these materials may be due to their covalent bonding as predicted below. The Cauchy pressure^35^ *C*_12_−*C*_44_ describing the angular character of a compound has also been computed. Negative value of Cauchy pressure indicates that in conjunction with angular character the material has directional (covalent) bonding too while the positive value shows the metallic bonding. In the present study, Cauchy pressure predicted for all lutetium monopnictides are negative which reflects their covalent character. The Cauchy pressure can also be related to the brittle/ductile nature of material. A material with negative value of Cauchy pressure is expected to be brittle whereas a positive value displays the ductile behaviour. From the results presented in Table 2, we found that the lutetium monopnictides show brittle character. To further support this point, we also calculated the Pugh’s ratio (*G/B*). According to Pugh’s criteria^36^, if *G/B* is larger than 0.57, the material behaves in a brittle manner, smaller values of Pugh’s ratio than 0.57 indicates ductile nature of a material. It is clear from Table 2 that the LuX compounds have *G/B* ratio greater than 0.57 which also supports their brittle character. Frantsevich *et al*.^37^ devised a rule on the basis of Poison’s ratio to distinguish between the ductility and brittleness of a material and fix a criteria that *v* should be less than 0.26 for a material to be brittle. The results obtained for *v* further assures the brittle character of LuX.

The Poisson’s ratio may also be used to obtain the information about the nature of bonding in a material. The value of Poisson’s ratio varies from 0.0 to 0.50 for different types of bonding. Covalently bonded materials have small value for *v* 33, for ionic crystals the critical value is 0.25^38^ whereas for metallic materials it is 0.33^38^. In present study, the value of *v* was found to be less than the critical value (0.25) also signifying the covalent nature of LuX.

Furthermore, the melting temperature of lutetium monopnictides has also been calculated employing the empirical relation (Eqn-9) proposed by Fine *et al*.^39^. The results obtained are listed in Table 3. It is observed that the predicted temperature is maximum for LuN and the melting temperature decreases as the mass of pnictide ion increases.


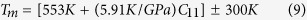


The longitudinal and transverse wave velocities along several crystallographic directions for these materials were also computed employing equations 9–14^40^. Here, *ρ* is the computed mass density. In the case of [110], the displacement of particles are along [1
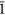
0] direction and perpendicular to K vector for shear velocity *v*_*s*_ whereas for longitudinal wave velocity *v*_*l*_, the displacement is along [110] direction and parallel to the K vector. The wave velocities calculated along several crystallographic directions are listed in Table 3.


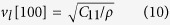



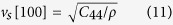



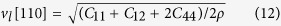



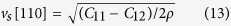



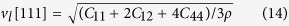



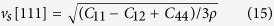


### Phonon Properties

**Figure 1:**
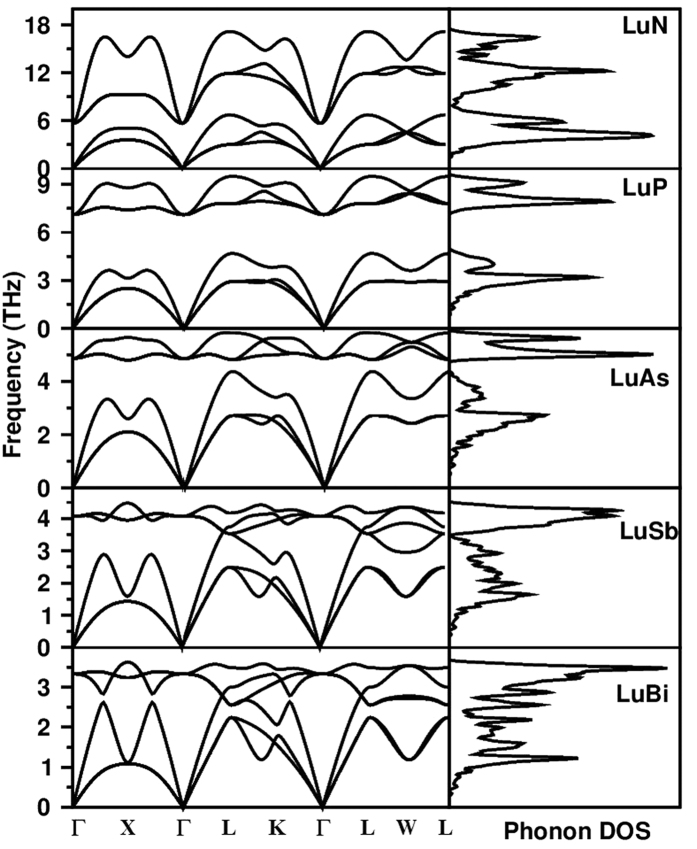
The calculated phonon dispersion curves and phonon density of states of lutetium monopnictides in NaCl-type structure using GGA+U approach.

The dispersion of phonons is an interesting property to understand the stability of a crystal besides knowing their thermal behaviour, superconductivity, Raman and thermal spectroscopy. Figure 1 shows the phonon dispersion curves (PDCs) along with phonon density of states (DOS) for lutetium monopnictides in NaCl-phase obtained using GGA+U approach. Since, we found that there was not a significant change in the elastic constants calculated with and without SOC, therefore, the phonon dispersion has been calculated without spin orbit coupling. Phonon frequencies calculated were found positive throughout the Brillouin zone which indicates the dynamical stability of these compounds. A frequency gap between the acoustical and optical phonon modes is apparent for LuP and LuAs whereas no such gap has been observed for LuN, LuSb and LuBi. For LuN, it is clear from Fig. 1 that the frequency of acoustic modes along the symmetry directions is larger than the frequency of optical modes at the symmetry point (Γ) which can be clearly seen from DOS as there is no frequency gap in this case. It can be also seen from Fig. 1 that the transverse optical (TO) and longitudinal optical (LO) modes are degenerate at Γ point only whereas TO — LO splitting can be seen at L, X and K symmetry points of the Brillouin zone. Also, transverse acoustic (TA) phonon modes are found to be nondegenerate along the Γ — K and K — L symmetry direction only. Besides, we observe that the longitudinal acoustic (LA) phonon modes meet the optical phonon modes along Γ — L symmetry directions for LuSb and LuBi. A large dispersion has been observed in optical modes for LuN whereas LuAs, LuSb and LuBi show large dispersion in acoustic phonon mode. The calculated optical zone centre frequencies were 5.66, 7.11, 4.85, 4.06, and 3.32 THz for LuN, LuP, LuAs, LuSb and LuBi respectively. We found that the highest observed optical frequency was largest for LuN and the frequency decreases as the mass of the pnictide ion increases.

## Conclusions

The structural, elastic and phonon properties of lutetium monopnictides have been studied using *ab initio* calculations employing GGA functional in the framework of DFT+U approach. The SOC interactions have been included to correctly predict the structural and elastic properties. Our results indicate that all lutetium monopnictides are mechanically stable and elastically anisotropic. The mechanical properties such as Young’s modulus *E*, Poisson’s ratio *v*, shear modulus *G*, and Pugh’s ratio have also been computed. From these results, we predict that all LuX compounds are brittle in nature and possess directional bonding. The melting temperature calculated is maximum for LuN and was found to decrease as the mass of pnictide ion increases. It is also found that the longitudinal wave velocities are larger than the shear wave velocities along the given directions for all lutetium monopnictides. The phonon frequencies of all lutetium monopnictides have been found positive throughout the Brillouin zone which indicates that these compounds are dynamically stable. A frequency gap between acoustic and optical phonons has been observed for LuP and LuAs only whereas no such gap was shown by LuN, LuSb, and LuBi.

## Methods

The structural property calculation of LuX (X = N, P, As, Sb, and Bi) have been performed within the framework of density functional theory using Vienna *ab initio* simulation package (VASP)^41^,^42^. We used spin-polarized formalism in the Perdew, Burke, and Ernzerhof (PBE) parameterization for the exchange and correlation functional^43^. The interaction between electrons and ion cores was described by projector-augmented wave method (PAW)^44^. The GGA+U^45^ method has been employed to take into account the self-interaction and strong localization of *f*-orbitals in LuX compounds. In this formalism, the Hubbard repulsion term U and the exchange term J were represented by single parameter U_eff_ = U–J. The value assumed for U_eff_, now onwards called as U only has been taken from ref. 46. The wave functions were expanded in a plane-wave basis set up to a kinetic energy cutoff of 600 eV. We have checked the energy cutoff convergence. Going from a cutoff of 600 eV to 900 eV, does not change the results. The integration within the Brillouin zone was performed with 9 × 9 × 9 grid using Monkhrost-Pack scheme^47^. We have checked the convergence in k-points. Going from 9 × 9 × 9 to 11 × 11 × 11 even to 15 × 15 × 15 does not change the results. The atomic geometry of the system obtained was fully relaxed until the Hellmann-Feynman forces exerting on all atoms were less than 0.005 eV/Å.

The elastic tensor was calculated by performing six finite distortions (in the order of 0.015 Å) of the lattice. Elastic constants were obtained then from strain-stress^48^ relationship with and without spin orbit coupling effect. Afterwards, the lattice dynamical properties were calculated by using finite displacement method as implemented in Phonopy^49^,^50^ with the support of VASP. The VASP interface was used to calculate the force constant matrix.

## Additional Information

**How to cite this article**: Mir, S. H. *et al*. Static and Dynamical Properties of heavy actinide Monopnictides of Lutetium. *Sci. Rep*. **6**, 29309; doi: 10.1038/srep29309 (2016).

## Figures and Tables

**Table 1:** The lattice constant (Å) and Bulk modulus (GPa) of Lutetium monopnictides calculated using GGA+U approach.

Property	LuN	LuP	LuAs	LuSb	LuBi
a	4.72	5.51	5.68	6.08	6.23
Expt.	4.76^a^	5.53^b^	5.68^c^	6.04^c^	6.16^b^
Theory	4.77^d^	5.53^d^	5.68^e^	6.09^d^	6.24^d^
*B*	171.84	89.36	79.91	61.73	54.72
Expt.	–	–	85 ± 3^c^	53 ± 4^c^	–
Theory	164.07^d^	87.24^d^	82.40^e^	60.69^d^	54.08^d^

^a^Ref. 40.

^b^Ref. 41.

^c^Ref. 8.

^d^Ref. 16.

^e^Ref. 5.

**Table 2:** The elastic constants of lutetium monopnictides calculated using GGA+U approach with SOC.

Compound	C_11_	C_12_	C_44_	A	G	E	ν	G/B	C_12_-C_44_
LuN	−(360.56)	−(77.48)	−(149.53)	(1.06)	(146.30)	(341.88)	(0.17)	(0.85)	(−72.05)
LuP	215.15 (217.05)	25.85 (25.52)	52.45 (52.53)	0.55	66.96	160.73	0.20	0.75	−27.01
LuAs	188.58 (191.94)	25.03 (23.59)	43.74 (43.58)	0.52	56.90	137.89	0.21	0.71	−19.99
LuSb	149.88 (150.22)	17.88 (17.48)	26.58 (25.91)	0.39	38.17	94.94	0.24	0.62	−8.43
LuBi	126.63 (129.75)	17.28 (17.21)	21.11 (22.22)	0.39	32.57	81.53	0.25	0.59	−5.0

Values in the brackets are results obtained without SOC. Shear modulus (*G*), Young’s modulus (*E*), Cauchy pressure (*C*_12_−*C*_44_) in *GPa*. The elastic anisotropy (*A*), Poisson’s ratio (*v*) and Pugh’s (*G*/*B*) ratio are dimensionless quantities.

**Table 3:** The mass density *ρ* (Kg/m^3^), longitudinal and shear wave velocities *V*_*l*_, *V*_*s*_ (Km/s), and melting temperature *T*_*m*_ (K) of lutetium monopnictides.

	*ρ × 10*^*3*^	*V*_*l*_*[100]*	*V*_*s*_*[100]*	*V*_*l*_[110]	*V*_*s*_[110]	*V*_*l*_[111]	*V*_*s*_[111]	T_m_ ± 300 K
LuN	12.01	5.48	3.53	5.54	3.43	5.56	3.46	2683.91
LuP	8.21	5.14	2.53	4.60	3.42	4.40	3.45	1835.76
LuAs	9.08	4.59	2.53	4.08	3.04	3.89	2.79	1687.36
LuSb	8.79	4.13	2.19	3.53	2.77	3.31	2.45	1440.80
LuBi	10.60	3.50	1.43	3.00	2.30	2.82	2.06	1319.82
