# Corrigendum: Static and Dynamical Properties of heavy actinide Monopnictides of Lutetium

**DOI:** 10.1038/srep32051

**Published:** 2016-08-24

**Authors:** Showkat H. Mir, Prakash C. Jha, M. S. Islam, Amitava Banerjee, Wei Luo, Shweta D. Dabhi, Prafulla K. Jha, R. Ahuja

Scientific Reports 6: Article number: 2930910.1038/srep29309; published online: 07 07 2016; updated: 08 24 2016

The original version of this Article contained a typographical error in the spelling of the author Amitava Banerjee, which was incorrectly given as Amitava Banarjee. This has now been corrected in the PDF and HTML versions of the Article.

